# Using protein complexes to predict phenotypic effects of gene mutation

**DOI:** 10.1186/gb-2007-8-11-r252

**Published:** 2007-11-27

**Authors:** Hunter B Fraser, Joshua B Plotkin

**Affiliations:** 1 Broad Institute of Harvard and MIT, 320 Charles St, Cambridge, Massachhusetts 02142, USA; 2 Department of Biology, University of Pennsylvania, 433 S. University Ave, Philadelphia, Pennsylvania 19104, USA

## Abstract

**Background:**

Predicting the phenotypic effects of mutations is a central goal of genetics research; it has important applications in elucidating how genotype determines phenotype and in identifying human disease genes.

**Results:**

Using a wide range of functional genomic data from the yeast *Saccharomyces cerevisiae*, we show that the best predictor of a protein's knockout phenotype is the knockout phenotype of other proteins that are present in a protein complex with it. Even the addition of multiple datasets does not improve upon the predictions made from protein complex membership. Similarly, we find that a proxy for protein complexes is a powerful predictor of disease phenotypes in humans.

**Conclusion:**

We propose that identifying human protein complexes containing known disease genes will be an efficient method for large-scale disease gene discovery, and that yeast may prove to be an informative model system for investigating, and even predicting, the genetic basis of both Mendelian and complex disease phenotypes.

## Abstract 2

The best predictor of a protein's knockout phenotype is shown to be the knockout phenotype of other proteins that are present in a protein complex with it.

## Background

Since the advent of genetic mapping, the approximate genomic locations of the polymorphisms that cause thousands of human phenotypes have been reported. As compiled by the Online Mendelian Inheritance in Man (OMIM) database, more than 1,500 human genes have been found to be associated with over 3,000 disorders [1]. This impressive level of success is tempered by the fact that more than 1,000 disorders have been mapped to a genomic region, but the underlying 'disease gene' has not yet been identified for these disorders [1]. Although some fraction of these 1,000 loci are surely false positives, the statistical significance associated with them indicates that most are likely to contain true Mendelian disease genes that have yet to be pinpointed.

This set of mapped disease loci represents an exciting opportunity for rapid advancement in our understanding of human disease genetics. Any method that can generate high-confidence predictions for which genes within the mapped regions are responsible for the diseases in question would be an important step forward. Indeed, some such methods were recently proposed, for example genomic screens for mitochondria-related genes identified several candidate disease genes for mitochondrial disorders [2,3].

Another route to gaining insights into a particular disease is to study a model of the disease in a nonhuman organism. Such models, if they are faithful reproductions of a specific human disease, can be informative by revealing aspects of the function of both wild-type and mutant versions of the disease gene (or its ortholog in the model organism) and by providing a testing ground for potential therapies. The mouse has been a particularly useful model in this regard. In general, the more diverged a model organism is from human, the more difficult it is to create an accurate model of a human disease; more deeply divergent lineages are less likely to have human disease gene orthologs, and they are also less likely to have a phenotype similar enough to humans to allow detailed study of a particular disease phenotype.

It is unfortunate that most diseases cannot accurately be modeled in species such as the bacterium *Escherichia coli *or the budding yeast *Saccharomyces cerevisise*, considering the ease of growing, storing, manipulating, and studying these organisms. Indeed, largely because of the simplicity of genetic manipulation in yeast, more functional genomic data have been generated for this species than for any other. For most genes/proteins, the mRNA expression level is known in thousands of conditions, as are the protein subcellular localization, the mRNA and protein decay rate, the mRNA translation rate, the protein abundance, the growth rates of systematic knockout strains across many conditions, a substantial fraction of the physical and genetic interactions, and much more.

Despite the vast amount of published functional genomic data, yeast and other unicellular organisms generally lack a morphologic phenotype rich enough to allow for detailed phenotypic descriptions based on a single growth condition. For example, even though different yeast strains may have distinct differences in their size, shape, and growth rate, in general very little information can be gleaned about a gene's knockout phenotype by observing growing cells in a single environment. However, if multiple environments are utilized in defining the phenotype, then even just one characteristic (such as growth rate) can be used to describe the phenotype with greater specificity, limited only by the diversity of environments tested. The description of a phenotype is simply a list of growth rates (or other measured characteristics) in all conditions tested, and two genes can be said to cause the same knockout phenotype if strains deleted for each gene exhibit similar growth rates across all tested environments. It is worth noting that this definition of phenotype is analogous to human disease, because any disease is simply a specific phenotype of lowered fitness in some set of environments.

The concept of identifying genes whose mutation or deletion leads to similar phenotypes is by no means novel. Indeed, much of classical genetics is based on this idea. Since the development of nearly comprehensive gene knockout or RNA interference knockdown resources in yeast, *Caenorhabditis elegans*, and *Drosophila melanogaster*, many researchers have systematically measured various phenotypes and identified clusters of genes with similar phenotypic profiles across a set of conditions [4-6].

Given such a phenotypic profile, we can ask what other types of data best predict 'phenotype pairs', that is, pairs of genes whose loss leads to similar phenotypic profiles. If we could identify an effective predictor of phenotype pairs, and if the predictor is sufficiently generic to apply to other species, then this predictor may be useful for elucidating human disease phenotypes as well. If this predictor has been measured for at least some human gene pairs, then it could then be used to predict human disease genes simply by searching the genome for gene pairs that score highly and for which one of the two genes is a known disease gene. For example, if co-expressed genes in yeast were found to be the best predictor of phenotype pairs, then it stands to reason that co-expressed human genes may also lead to the same phenotype when mutated. If so, then identifying all genes that are co-expressed with a known disease gene would give a list of candidates for additional genes that cause the same disease. By combining the candidate list with mapped but unidentified disease genes, more confidence could be given to candidate genes that fall within the mapped susceptibility loci. In this manner, genes that are likely to be responsible for any type of disease could be identified, as long as the disease has at least one known causative gene. Others have previously used physical interactions between proteins or multiple gene-ranking algorithms to predict new disease genes from within mapped susceptibility loci [7-9]. However, because the functional genomic data for humans is currently rather sparse (compared with what is available for some model organisms), it remains to be seen whether some type of data not yet explored in humans could be even more predictive of human disease genes.

To discover what predictor(s) might be the most effective in human, we turned to yeast as a model. We reasoned that if quantitative phenotypes can be studied in yeast, then the vast amount of functional genomic data available could be used to predict the phenotypic effects of gene mutations or deletions. Here, we utilize a general framework for studying phenotypes to find what types of data are predictive of phenotypes in yeast; we then apply this framework to human disease phenotypes.

## Results

### Protein complexes as predictors of phenotype

Several groups have noted that subunits of the same protein complex tend to have similar knockout/knockdown phenotypes in both yeast and *C. elegans *[4,5,10]. However, other potential predictors of phenotype pairs were not compared with protein complexes, so from these studies it is not possible to conclude what type of information is the best available predictor.

To address this issue, we utilized the most comprehensive phenotypic profiling dataset published to date: quantitative growth rates of the yeast haploid deletion collection, including more than 4,200 strains, in 82 diverse conditions [6]. The conditions include seven crude antifungal extracts, 23 US Food and Drug Administration approved drugs, and more than 50 other synthetic compounds. The original authors did not attempt to test any predictors of phenotypic profile similarity. In this dataset, we defined a 'phenotype pair' as a pair of genes whose knockout strains have growth rate correlation coefficient *r *> 0.8 across all 82 conditions. This definition resulted in approximately one phenotype pair for every 4,000 pairs of genes.

We compiled a list of 20 potential predictors of phenotype pairs in yeast. The list included genetic interactions compiled from the literature [11]; pairs of genes sharing a Pfam [12] protein domain or bound by the same transcription factor [13]; mRNA co-expression at several correlation cut-offs using a large compendium of expression profiles [14]; protein co-localization measured with a collection of green fluorescent protein tagged strains [15]; co-citation in the literature [16]; similar phylogenetic profiles (that is, pattern of ortholog presence and absence across species) [16]; all known yeast metabolic pathways [17]; several datasets of physical interactions and protein complexes, either from high-throughput (HTP) screens or from the literature [10,11,18,19], and two published classifications of gene 'modules' or functional relationships defined using multiple data sources [16,20].

We then devised a test to use each of these data types as a separate predictor of phenotype pairs. We did not wish the test to penalize data types that cover fewer pairs of genes than others (for example, most gene pairs have not been tested for genetic interactions, so this data type has low coverage); therefore, our metric of predictive success was simply the enrichment for phenotype pairs within the set of gene pairs satisfying the criterion used. For example, gene pairs in the same metabolic pathway form one set of predictions; another set consists of genes co-expressed with correlation coefficient *r *> 0.3 in our expression compendium. The enrichment within that set is the number of phenotype pairs found within that set, divided by the number expected by chance. Enrichments greater than one indicate more predictive power than random.

**Figure 1:**
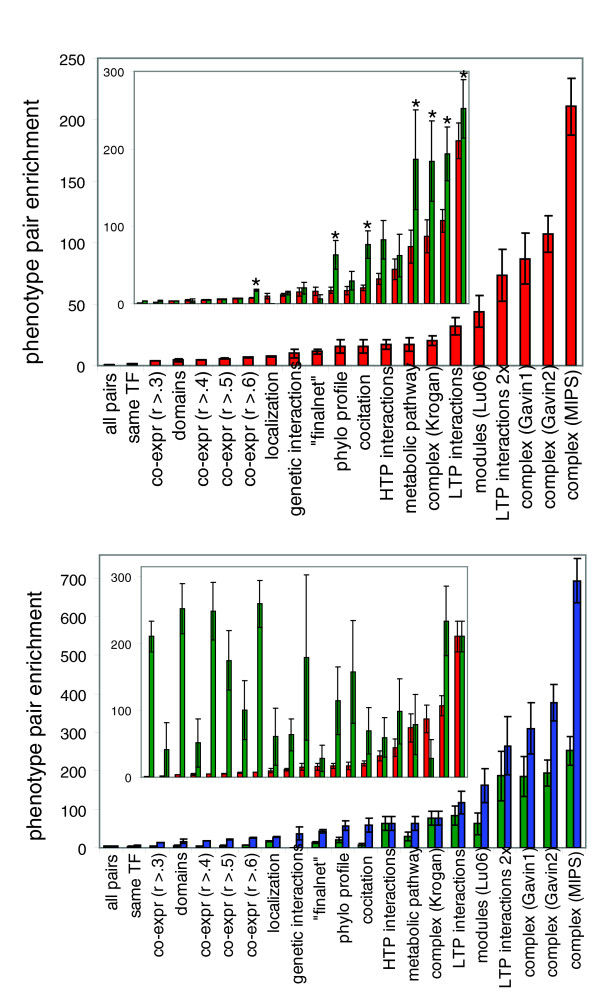
Predictors of phenotype pairs in yeast. **(a) **Enrichments for phenotype pairs among 20 predictors. An enrichment value of 1 reflects random performance (shown as 'all pairs', the left-most column) and greater than 1 indicates better than random predictive power. Predictors are arranged in order of increasing predictive power. Error bars indicate the hypergeometric standard deviation, which reflects the range of expected variation in the enrichment value. In the inset, red bars are the same as in the main panel a, and are in the same order. Green bars indicate enrichments in the intersection of each dataset with co-expression (*r *> 0.3). The seven datasets with significant improvements in predictive power are indicated by asterisks. Note that the four co-expression datasets are not counted in the multiple testing correction because they cannot possibly show any improvement when intersecting with another dataset that is a superset. **(b) **Green bars are the same as in Figure 1a inset. Blue bars indicate the level of enrichment that would be expected by chance, if co-expression was entirely independent of each dataset. Green bars significantly lower than the paired blue bar indicate a dataset that is not independent of co-expression. Error bars indicate the hypergeometric standard deviation, which reflects the range of expected variation in the enrichment value. In the inset, red bars are the same as in panel a and are in the same order as in both panels a and b. Green bars indicate enrichments in the intersection of each dataset with Munich Information Center for Protein Sequences (MIPS) complexes. Note that although many green bars are significantly higher than the paired red bars, no green bars are significantly higher than the MIPS complexes (rightmost) bars. This indicates that no dataset adds to the predictive power of complexes among the set of proteins in MIPS complexes. HTP, high-throughput; LTP, low-throughput; TF, transcription factor.

Testing the enrichment for all 20 predictors, we found a striking pattern (Figure 1a); whereas 19 of the predictors gave at least a twofold enrichment for phenotype pairs over the random expectation (*P *< 10^-6 ^for all 19), some yielded much greater enrichments than others. The three datasets with greater than 80-fold enrichment all consisted of protein complexes: two different metrics of stable protein interactions from a recent HTP screen [10] (see Materials and methods, below), and the set of high-confidence manually curated protein complexes from the Munich Information Center for Protein Sequences (MIPS) database [19]. In fact, all seven predictors with greater than 20-fold enrichment (Figure 1a) were protein complexes, physical interactions compiled from the (non-HTP) literature [11], or 'modules' of co-expressed proteins with many physical interactions among themselves [20]. Considering that both the physical interactions compiled from the literature and the 'modules' [20] are expected to be highly enriched for protein complexes, it appears that the best predictors are united by the theme of stable protein interactions.

We next sought to test whether combining different datasets by taking their intersection might improve their predictive power. For example, we could ask whether gene pairs that are co-expressed and that also have similar phylogenetic profiles are more enriched for phenotype pairs than are either of these two predictor datasets alone. If the set of pairs matching both criteria has a significantly higher frequency of phenotype pairs than pairs matching either one of the two criteria alone, then we can conclude that the two data sets contain independent information; in other words, each dataset contains some information that is not present in the other. If, instead, the intersection yields an enrichment that is not greater than the enrichment from either criterion alone, then there is no evidence of independent information.

We first measured the predictive power of the intersections between each of the 20 predictors and co-expression. We found that intersecting with co-expression significantly (*P *< 0.01 after Bonferroni correction for multiple tests) improved the enrichment for phenotype pairs in seven datasets, and it did not significantly diminish the enrichment for any dataset (Figure 1a [inset], compare enrichments before [red] and after [green] intersection; asterisks indicate significant improvement). Aside from these seven significant improvements, no dataset scored better than *P *= 0.37 for improvement in predictive power, indicating a clear distinction between the datasets improved by intersecting with co-expression and those not improved. Interestingly, six of the seven improved datasets consisted of physical interactions (the seventh was protein co-localization): three lists of HTP protein complexes from two studies [10,18], one list of all published HTP physical interactions (excluding the two HTP protein complex screens treated separately here) [11], and two lists (with different confidence levels) of all physical interactions from non-HTP publications [11]. Because protein complex subunits tend to be tightly co-expressed with one another [21], one possible interpretation of this result is that intersecting physical interactions with co-expression improves enrichments by reducing false-positive results (not expected to be uncommon among some HTP screens, as well as among non-HTP interactions reported only once in the literature) and/or by decreasing the frequency of transient interactions (which comprise many of the interactions in the three noncomplex interaction datasets). One prediction of this idea is that for a dataset consisting of protein complexes with very few false positives, intersecting with co-expression should not increase the predictive power. Consistent with this idea, the high-confidence MIPS complexes are the only physical interaction data that were not significantly improved by intersecting with co-expression (Figure 1a [inset]; *P *= 0.66). As with Figure 1a, all of these results are consistent with protein complexes being the key predictors of phenotype pairs.

It is informative also to compare enrichments for phenotype pairs seen in Figure 1a (inset) with what would be expected by chance, if each dataset were entirely independent of co-expression (two predictors are independent if the size of their intersection, both within the set of phenotype pairs and within the set of nonphenotype pairs, is no greater than expected by random chance). The expected enrichment for phenotype pairs within an intersection of two independent criteria is a simple function of the frequencies of phenotype pairs satisfying each criterion alone, and the background frequency of all phenotype pairs (see Materials and methods, below). Comparing these expected enrichments (Figure 1 [blue bars]) with the observed intersection enrichments (Figure 1b [green bars]), it is clear that in many cases the observed enrichment is close to that expected under independence, indicating that co-expression is adding nearly orthogonal information. In no case, however, is there a significant increase over the expectation assuming independence. In summary, for a number of the datasets (in particular, the six for which intersecting with co-expression significantly improves the predictive power), the information added by intersecting with co-expression is close to what would be expected if co-expression contained entirely independent information about phenotypes.

In stark contrast to co-expression, when intersecting the set of MIPS complexes with all other datasets, there was no improvement for phenotype pair enrichment above the enrichment found in MIPS complexes alone. This is shown in Figure 1b (inset), in which the observed phenotype pair enrichments (green bars) can be compared with MIPS complexes alone (the rightmost variable); although four intersections give slightly higher enrichments than MIPS complexes, the improvement is not significant in any case. In sum, no dataset tested here adds information about phenotypes when we control for protein complexes, even though nearly every predictor does have a significant level of predictive power on its own. This is exactly what would be expected if all datasets were predictive largely because they are themselves enriched for members of the same complexes.

We next tested whether the intersection between any two of our datasets had greater predictive power than MIPS complexes alone. Strikingly, not a single intersection (out of all 190 combinations) gave a significant improvement over MIPS complexes alone (not shown). The most predictive combination that did not include complexes as one of the predictors was the intersection of co-expressed pairs with the high-confidence literature-derived physical interaction data (an intersection that is itself highly enriched for protein complexes), which enriched for phenotype pairs 186-fold over random (Figure 1a [inset]) at co-expression *r *> 0.3. At more extreme co-expression cut-offs, the enrichment increased even more (up to 310-fold at *r *> 0.6), although it was never significantly better than MIPS complexes alone. These results indicate that even in the absence of a reliable protein complex membership list, phenotype pairs can be effectively predicted by using a proxy for protein complex membership.

### Predicting human disease genes

Having established that protein complexes are the most powerful predictor of phenotype pairs in yeast, we reasoned that this property might apply to other species as well. Two general lines of evidence support this idea. First, other general properties that characterize relationships between genes or proteins (for example, that subunits of the same protein complex are often co-expressed [21]) are usually conserved between species. Second, there is evidence that subunits within each of 11 well characterized protein complexes in *C. elegans *exhibit similar RNA interference knockdown phenotypes [5], as well as anecdotal evidence that subunits of the same complex can sometimes cause the same human disease (for instance, Fanconi anemia [22] and limb-girdle muscular dystrophy [23]).

We therefore sought to test systematically how best to predict human phenotype pairs. For human, we define a phenotype pair in a similar although less quantitative manner as for yeast: a pair of genes whose mutation leads to a similar phenotype. Similar disease phenotypes were compiled from the OMIM database [1] and grouped into clusters, as described previously [7], resulting in a list containing approximately one out of every 26,000 gene pairs.

Because the range of human functional genomic data lacks the breadth of published yeast data, it is not possible to compare a large number of human phenotype pair predictors. In particular, only a very small number of protein complexes have been characterized, and so we were unable to test directly whether complexes enrich for phenotype pairs to the same extent as in yeast. Furthermore, transferring MIPS complexes by orthology (assuming that all human orthologs of yeast MIPS complex subunits have conserved interaction partners) does not result in a large enough list of putative interactions to be informative (not shown).

However, there do exist human gene expression data from thousands of conditions, as well as tens of thousands of known physical interactions. Considering how well the co-expressed literature-derived interactions predicted phenotype pairs in yeast (Figure 1a [inset]), we decided to use co-expressed physical interactions as a proxy for protein complexes, with the understanding that this list is likely to contain a large number of noncomplex pairs.

We assembled several human datasets for this analysis. To calculate co-expression, we used a compilation of 2,642 Affymetrix U133a microarrays (see Materials and methods, below). We also used two physical interaction datasets: literature-derived non-HTP human interactions from the Human Protein Reference Database (HPRD) database [24] and HTP interaction data from both human [25,26] and other species whose interactions were mapped to human by orthology [7].

**Figure 2:**
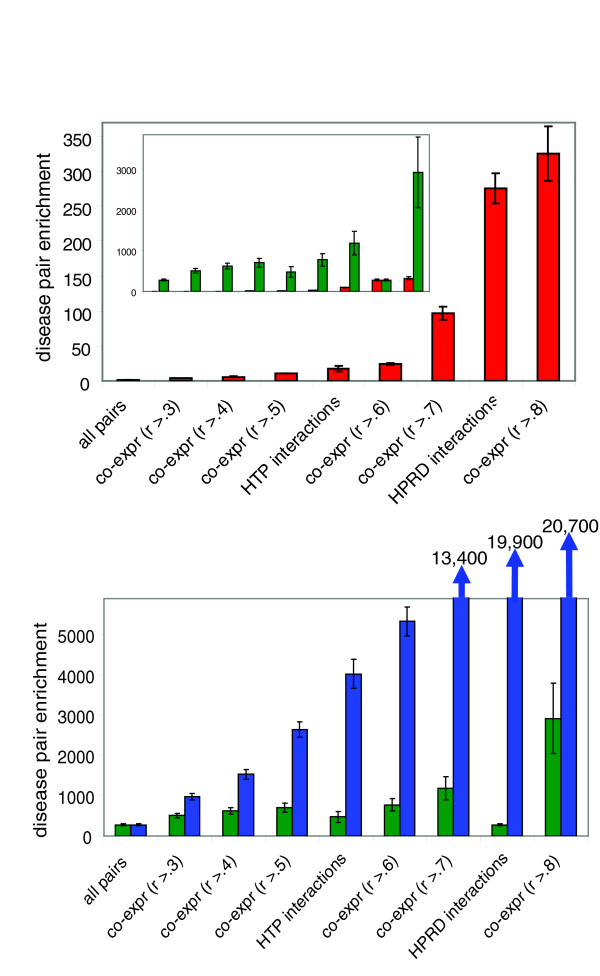
Predictors of disease gene pairs in human. **(a) **Enrichments for disease gene pairs among eight predictors. An enrichment value of 1 reflects random performance (shown as 'all pairs', the left-most column). Predictors are arranged in order of increasing predictive power. Error bars indicate the hypergeometric standard deviation, which reflects the range of expected variation in the enrichment value. In the inset red bars are the same as in the main panel a and are in the same order (note the tenfold change in scale). Green bars indicate enrichments in the intersection of each dataset with Human Protein Reference Database (HPRD) interactions. Aside from HPRD intersected with itself or with all pairs, all but one dataset (high-throughput [HTP] interactions) exhibit a significant improvement in predictive power over HPRD interactions alone when intersected with HPRD; this indicates that these datasets are at least partially independent of HPRD. **(b) **Green bars are the same as in panel a (inset). Blue bars indicate the level of enrichment that would be expected by chance, if HPRD interactions were entirely independent of each dataset. Green bars significantly lower than the paired blue bar indicate a dataset that is not entirely independent of HPRD interactions. The three right-most blue bars are truncated for clarity; their enrichment values are written above each bar. Error bars indicate the hypergeometric standard deviation, which reflects the range of expected variation in the enrichment value.

In agreement with previous results [7], we found that the HPRD interactions (271-fold above random) were far more predictive of phenotype pairs than were HTP interactions (17-fold above random; Figure 2a). Co-expression was a relatively weak predictor at a wide range of correlation cutoffs (for example, 3.7-fold above random at *r *> 0.3); at high thresholds, however, co-expression equaled or slightly exceeded the HPRD interactions in predictive power (325-fold enrichment at *r *> 0.8). All of these predictors gave highly significant (*P *< 10^-8^) improvements over random pairs.

As was the case in yeast, taking the intersection of physical interactions and co-expression dramatically improved predictive power. For the HTP interactions, the enrichments improved to 40-fold above random by intersecting with co-expression *r *> 0.3 and 43-fold with *r *> 0.5 (at higher co-expression cut-offs no disease pairs were present among the HTP interactions). Taking the intersection of co-expression with HPRD interactions resulted in an even better predictor of disease gene pairs: approximately 500-fold above random at *r *> 0.3 and 3,000-fold at *r *> 0.8 (Figure 2a [inset]). This impressive approximate 3,000-fold enrichment results in 11% (10/92; listed in the Additional data file 1) of all gene pairs satisfying these criteria being pairs known to cause the same disease. We note that 11% may be an underestimate, because many physical interactions and disease genes are yet to be discovered; alternatively it may be an overestimate, because of biases in the scientific literature (see Discussion, below). All of the intersections with HPRD interactions had significantly (*P *< 10^-4 ^after Bonferroni correction for seven tests) less enrichment than expected by chance under independence (Figure 2b), indicating that neither co-expression nor HTP interactions are completely orthogonal to HPRD interactions. In fact, we found that much of the information in the HTP data is redundant with HPRD, because this intersection was the only one with no significant improvement over HPRD alone (*P *= 0.17).

Considering the magnitude of the enrichment among co-expressed literature-based interactions for pairs of genes involved in the same disease, it is possible to begin to make predictions about novel disease genes. For example, among our current predictions are six genes (*COL4A1*, *COL4A2*, *SPARC*, *BGN*, *DCM*, and *LUM*) whose mutation may lead to phenotypes similar to Ehlers-Danlos syndrome (which is characterized by a range of problems related to skin, joints, eyes, and other areas), based on their co-expression and physical interactions with three proteins known to be involved in this disease (*FN1*, *COL3A1*, and *COL1A2*). Other predictions include involvement of *MCM2 *and *MCM3 *in hypolactasia, *S100B *in Alexander disease, and *CFHL1 *in chronic hypocomplementemic nephropathy. These predictions, albeit few in number, serve to illustrate how a large-scale protein complex membership list could be used to predict a much greater number of novel human disease genes.

## Discussion

We have shown that protein complexes appear to be the most effective predictors of similar phenotypic effects for gene pairs. Despite the myriad types of functional and evolutionary genomic data we tested, no dataset was able to increase the predictive power of complexes alone. Furthermore, all of the most effective predictors of yeast phenotype pairs were either protein complexes (Figure 1a) or co-expressed physical interactions (Figure 1a [inset]), which are themselves highly enriched for complexes. Applying this idea to human data, we found that co-expressed physical interactions are effective predictors of gene pairs known to cause the same disease (Figure 2a [inset]). This indicates that previous studies that used only protein interactions to predict disease genes [7,9] might have greatly improved their predictive power by incorporating co-expression information as well.

One possible concern is that the literature-based interactions are not truly independent of the disease gene pairs. This situation could arise if investigators preferentially look for interactions between proteins that are known to be involved in the same disease, or if a protein's role in some disease was discovered (at least in part) as a result of its interaction with a known disease-related protein. Unfortunately, it is very difficult to control for this possibility. For example, if a protein interaction is discovered after both proteins involved have been found to cause the same disease, then one could in principle read the publication reporting the interaction to see if the authors cite the proteins' role in disease as a factor in their research. However, even if the relation with disease is not cited as a reason why the interaction was sought out, this does not rule out the possibility that the proteins' role in disease contributed in some way to the discovery of the interaction. In sum, conclusive evidence of either independence or dependence between the discovery of the proteins' interactions and their role in the same disease cannot usually be found.

Fortunately, however, the enrichments for human disease gene pairs that we observed are strong enough that even extreme biases would not be sufficient to account for all of the enrichment we observe. For example, if we were to find that only half of all pairs of genes causing the same Mendelian disease were known, and that among the other half not even a single pair involved a physical interaction, then our observed enrichments would be reduced by twofold. Our strongest enrichment (Figure 2a [inset]) would thus be reduced to about 1,500-fold over random, which is still a very useful level of enrichment for predicting disease gene pairs.

If protein complexes are an even better predictor of disease gene pairs than co-expressed physical interactions, as appears to be the case in yeast, then a high-quality human protein complex membership list could be even more predictive that than the approximate 3,000-fold enrichment we observed. For this reason, we propose that identifying human protein complexes may be the most efficient method for identifying the genes responsible for many mapped disease loci. Indeed, because of recent technologic advancements, identifying the subunits of human protein complexes is not difficult [27,28]; thousands of human open reading frames, cloned into Gateway vectors [29], can easily be tagged for affinity purification, transfected/infected into an appropriate human cell line, purified, and subjected to mass spectrometry to identify all proteins co-purifying with the tagged protein. The most promising candidates for this approach would be proteins that are known to cause a disease for which there are many mapped susceptibility loci with unidentified causal genes, because these present the best opportunity for discovering the causal genes residing within susceptibility loci. If a protein encoded by a gene within a mapped susceptibility locus is found to be in a protein complex with a known disease gene, then this prediction could be tested by sequencing the gene in the DNA samples used for the original genetic mapping study. Also, in addition to revealing novel disease genes, identifying the subunits of protein complexes containing disease-associated proteins may greatly improve our understanding of the biology underlying these diseases.

The general framework presented here could also be applied to more complex, multigenic disease phenotypes. For example, with a large enough set of unbiased genetic interactions from yeast, the same 20 predictors used here could be applied to identify the best predictor(s) of genetic interactions in yeast. These predictor(s) could then be used to predict epistatic interactions that are thought to be responsible for many complex diseases [30-32]. Indeed, such a method could be applied to any complex phenotype in any species, and could possibly aid in our general understanding of how genotypes determine phenotypes.

## Materials and methods

### Datasets

Yeast data were compiled from a number of sources. Expression data were from a compilation of 1,610 published microarrays [14], and co-expression was calculated as the Pearson correlation between pairs of genes across all experiments. Increasing the co-expression cut-off above *r *> 0.6 did not increase enrichments, so these cut-offs are not shown in Figure 1. Transcription factor binding sites [13] were required to have both binding site conservation in at least three out of four *Saccharomyces sensu stricto *spp. and 'ChIP-chip' (chromatin immunoprecipitation-chip) binding data at *P *< 0.005 in order to call a promoter as bound by a particular transcription factor. Pfam domains present in every yeast gene were downloaded from the Pfam database [12]. Co-localization data were from Huh and coworkers [15]; two proteins were called co-localized if they were present in exactly the same set of subcellular locations. Genetic interactions, HTP interactions, and literature-curated physical interactions were from Reguly and colleagues [11]. Phylogenetic profile similarity, co-citation, and 'finalnet' (a composite score calculated from many datasets) were taken from Lee and coworkers [16]; cut-off scores of 0.5, 2, and 3 were used for each dataset, respectively (altering cut-offs did not greatly affect the results). Metabolic pathways were taken from Forster and colleagues [17]. Functional 'modules' of genes were defined by Lu and colleagues [20] as co-expressed groups of proteins with many physical interactions among themselves. The four protein complex datasets were from three sources [10,18,19]. Two different datasets of interactions were provided by Gavin and coworkers [10]: a list of complexes ('Gavin1') and a socio-affinity score between pairs of proteins ('Gavin2'; cut-off = 5). For the MIPS complexes, we used all pairs of proteins present in the same complex, excluding the ribosome (since this single complex has more protein pairs than all others combined, so would be almost entirely responsible for any results we found). Raw growth rate data across 82 growth conditions were taken from [5]; a threshold of Spearman *r *> 0.8 was used to define pairs of genes whose knockout causes the same phenotype (all results were largely robust to changes in this threshold; in general, increasing the threshold resulted in stronger enrichments but smaller phenotype pair sample sizes, whereas decreasing the threshold resulted in weaker enrichments but larger sample sizes).

Human datasets were from two sources. For gene expression data, we chose Affymetrix U133a (Affymetrics Inc., Santa Clara, CA, USA) as the platform because this microarray has more raw data (2,642 CEL files) deposited in the Gene Expression Omnibus database [33] than any other (we did not attempt to combine data from multiple different microarray platforms, because doing so can be problematic [not shown]). CEL files were downloaded from Gene Expression Omnibus in August 2006, and Robust Multichip Average normalization [34] was performed (R Lee and B Hayete, personal communication). Co-expression values were calculated as the Pearson correlation between gene pairs. We obtained the other datasets from Oti and coworkers [7]: disease data, in which all diseases from the OMIM database [1] with known causative genes were grouped by similarity (see Oti and coworkers [7] for details); HTP physical interactions from both human [25,26] and from other species (*S. cerevisiae*, *C. elegans*, and *D. melanogaster*) transferred to human by orthology using the Inparanoid algorithm [7]; and non-HTP literature-based physical interactions from the HPRD database [24]. All human data were mapped to Ensembl genes [35] for analysis; if multiple Affymetrix U133a microarray probe sets matched a single gene, then their median value in each microarray was calculated before calculating co-expression.

### Statistics

All *P *values reported were calculated using the hypergeometric test for enrichment [36]. In all cases, this test was used to calculate whether a given set of gene pairs had a different frequency of phenotype/disease pairs than would be expected by chance, given the sample sizes involved and the expected frequency of such pairs. The expected random frequency depended on what was being tested. For example, to compare single predictors to random pairs, the expected frequency of phenotype/disease pairs was that of random pairs. To compare intersections of predictors to single predictors, the expected frequency was the greater of the two predictors alone. To compare intersections of predictors to the expectation under the assumption of independence, the expected frequency was given by the following equation:

$$e=\frac{(1-f_{1})f_{2}f_{3}}{f_{1}(1-f_{2})(1-f_{3})+(1-f_{1})f_{2}f_{3}}$$

Where *e *is the expected frequency by random chance, *f*_1 _is the frequency of phenotype/disease pairs among all pairs of genes, *f*_2 _is the frequency among gene pairs satisfying one of the criteria being used, and *f*_3 _is the frequency among gene pairs satisfying the other criterion.

## Abbreviations

HPRD, Human Protein Reference Database; HTP, high-throughput; MIPS, Munich Information Center for Protein Sequences; OMIM, Online Mendelian Inheritance in Man.

## Authors' contributions

HBF and JBP conceived of the analyses and wrote the paper. HBF performed the analyses. Both authors read and approved the final manuscript.

## Additional data files

The following additional data are available with the online version of this paper. Additional data file 1 is a table listing the top 92 predictions of gene pairs most likely to cause the same disease, as assessed by physical interaction in the HPRD database and co-expression.

## Supplementary Material

Additional data file 1 Presented is a table listing the top 92 predictions of gene pairs most likely to cause the same disease, as assessed by physical interaction in the HPRD database and co-expression. Click here for file
